# Bismuth Telluride and Its Alloys as Materials for Thermoelectric Generation

**DOI:** 10.3390/ma7042577

**Published:** 2014-03-28

**Authors:** H. Julian Goldsmid

**Affiliations:** School of Physics, University of New South Wales, Sydney 2052, Australia; E-Mail: hjgoldsmid@bigpond.com; Tel.: +61-362-291-776

**Keywords:** thermoelectric, generation, bismuth telluride

## Abstract

Bismuth telluride and its alloys are widely used as materials for thermoelectric refrigeration. They are also the best materials for use in thermoelectric generators when the temperature of the heat source is moderate. The dimensionless figure of merit, *ZT*, usually rises with temperature, as long as there is only one type of charge carrier. Eventually, though, minority carrier conduction becomes significant and *ZT* decreases above a certain temperature. There is also the possibility of chemical decomposition due to the vaporization of tellurium. Here we discuss the likely temperature dependence of the thermoelectric parameters and the means by which the composition may be optimized for applications above room temperature. The results of these theoretical predictions are compared with the observed properties of bismuth telluride-based thermoelements at elevated temperatures. Compositional changes are suggested for materials that are destined for generator modules.

## 1. Introduction

Since the observation [1], sixty years ago, of a substantial lowering of temperature by means of the Peltier effect in a thermocouple incorporating bismuth telluride, Bi_2_Te_3_, this compound has been extensively used in the construction of thermoelectric modules. The performance of these modules has steadily improved, since the original observations, due to a number of factors. The thermoelectric figure of merit [2], which nowadays usually appears in its dimensionless form, *ZT*, has increased from the order of 0.5 to values significantly greater than one. Here *Z* is defined as α^2^σ/λ, where α is the Seebeck coefficient, σ is the electrical conductivity and λ is the thermal conductivity. Strictly speaking, *Z* should be defined for a pair of thermoelements, since the Peltier and Seebeck effects are manifest for thermocouples rather than single materials, but it is convenient to select materials on the basis of the maximization of *Z* for each branch. It is only when the two branches have widely different properties that this strategy must be modified as, for example, when one branch is a superconductor [3,4].

From the outset it was realized that there was the need to optimize [1,2] the charge carrier concentration, by doping with donor or acceptor impurities. When there is only one type of carrier, electrons or positive holes, the Seebeck coefficient at a given temperature falls as the electrical conductivity increases. The Seebeck coefficient and the electrical conductivity are combined in a quantity, α^2^σ, known as the power factor. One aims to make this parameter as large as possible, though it must be remembered that a large electrical conductivity also implies a large electronic component of the thermal conductivity. Most of the early improvements came about through a reduction in the lattice component of the thermal conductivity, λ_L_. This was achieved through the use of solid solutions [5] of bismuth telluride with the isomorphous compounds antimony telluride and bismuth selenide. The enhanced scattering of phonons in these solid solutions is not usually accompanied by a reduction in the mobility of the charge carriers. This is somewhat surprising since the charge carriers usually possess the larger mean free path.

In recent years, further reductions in the lattice conductivity have been obtained by the adoption of nanostructures. Although the original aim seems to have been the improvement of the power factor through quantum confinement effects [6], in actual fact it appears that the main advantage has stemmed from phonon scattering on the boundaries of nano-sized grains [7,8]. In other words, nano-structuring usually seems to affect the lattice conductivity rather than the electronic transport properties.

It is important to realize that the same figure of merit applies for thermoelectric generation [2], using the Seebeck effect, as for refrigeration using the Peltier effect. Thus, at any given temperature, the best refrigeration material will also be the best material for generation. In so far as bismuth telluride alloys are the best materials at room temperature, they must also be the best generator materials, at least close to this temperature.

One must remember that the electrical and thermal conductivities of bismuth telluride are anisotropic [9] although the Seebeck coefficient does not depend on orientation in the extrinsic or one-carrier regime. The lattice conductivity is about twice as large along the cleavage planes as it is in the perpendicular direction [10]. The anisotropy of the hole mobility is almost the same as that of the lattice conductivity so that, although the electrical and thermal conductivities in aligned crystals are different from those in randomly oriented polycrystalline material, the figure of merit is virtually isotropic for p-type bismuth telluride. On the other hand, the electron mobility is more strongly anisotropic than the lattice conductivity and this means that the figure of merit is significantly less for non-aligned n-type samples [11] than it is for properly oriented material. This is unfortunate since there are practical advantages in making material in polycrystalline form using a sintering process rather than a melt-growth process. For example, the concept of a bulk nanostructure is probably most easily realized using sintered material. Unless some measure of orientation can be achieved during sintering, the advantage of a reduction in the lattice conductivity due to nanostructuring may be outweighed by a fall in the power factor, α^2^σ.

### 2. Effect of Temperature on the Thermoelectric Properties

#### 2.1. Single Carrier Conduction

Let us first discuss the behavior of a material with a single parabolic conduction or valence band. At any particular temperature there will be an optimum value of the Fermi energy, *E*_F_, and a corresponding optimum Seebeck coefficient. This will be determined predominantly by the need to maximize the power factor, but there will also be some influence from the electronic component, λ_e_, of the thermal conductivity, unless this quantity is negligibly small compared with the lattice component, λ_L_.

When the temperature is raised while the carrier concentration stays constant, the Fermi energy moves out of the band and into the energy gap [12]. The Seebeck coefficient eventually rises above its optimum value, a value that is more-or-less independent of temperature. Material intended for use at higher temperatures should have an increased carrier concentration to match the temperature- dependent Seebeck coefficient to the range of operation. This means that the dimensionless Fermi energy η, equal to *E*_F_/*kT*, will remain almost independent of temperature. Since the effective density of states 2(2πm*kT/h^2^)^3/2^ is proportional to *T*^3/2^, the carrier concentration for a fixed value of η is also proportional to *T*^3/2^. Also, if the carriers are scattered by acoustic-mode phonons, the mobility should vary as *T*^−3/2^. Thus, the optimum electrical conductivity for this simple model should not vary with temperature. This means that the optimum power factor α^2^σ should also be temperature independent. We note that α^2^σ/λ_e_ will decrease as the temperature rises, since λ_e_ is proportional to *T*, in so far as the Wiedemann-Franz law is applicable. However, *ZT* is equal to α^2^σ*T*/(λ_e_ + λ_L_) where λ_L_ is usually appreciably larger than λ_e_. Moreover, λ_L_ generally decreases with increasing temperature (the variation will vary from *T*^−1^ for a pure element or compound to *T*^0^ when alloy scattering is completely dominant [13]). Thus, the total thermal conductivity may be approximately independent of temperature since the temperature variations of the two components may just about balance out.

Let us consider a hypothetical optimized material that happens to have *ZT* equal to 1. We may write an approximate expression for *ZT* in terms of the ratio *C* of the lattice conductivity to the electronic thermal conductivity. The optimum Seebeck coefficient will be close to 2.5 (*k/e*) (*i.e.*, 216 μV/K). We suppose that the electronic thermal conductivity is equal to *A*(*k/e*)^2^σ*T*, where *A* is equal to about 2 for a conductor that is not strongly degenerate and for which acoustic-mode lattice scattering is predominant. The expression for *ZT* is then:

$$ZT\approx \frac{2.5^{2}}{2\left(C+1\right)}$$ (1)

Thus, if *ZT* is equal to about 1, *C* is equal to about 2.1. This means that in a typical optimized thermoelectric material the lattice conductivity is just over twice the electronic thermal conductivity.

**Figure 1.**
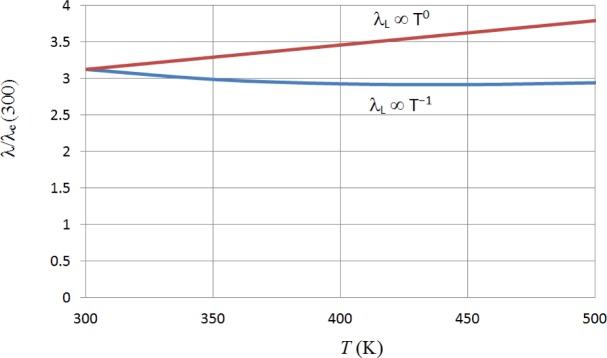
Plot of relative total thermal conductivity against temperature for λ_L_ independent of temperature (upper plot) and for λ_L_ inversely proportional to temperature (lower plot).

In Figure 1 we plot the total thermal conductivity, relative to the electronic thermal conductivity at 300 K, against temperature, for the two extremes of the lattice conductivity, *i.e.*, λ_L_ proportional to *T*^−1^ and *T*^0^. The electronic thermal conductivity has been calculated using Fermi-Dirac statistics [12]. It will be seen that the assumption that the thermal conductivity is more-or-less independent of temperature is likely to be a good approximation. Our overall expectation, then, is that *ZT* at the optimum Seebeck coefficient should rise with increasing temperature, perhaps being approximately proportional to *T*.

Our model is not quite valid for bismuth telluride and its alloys, even when the temperature is low enough for there to be only one type of charge carrier [14]. The carrier mobility in the non-degenerate region has been assumed to be proportional to *T*^−3/2^ whereas it is actually observed to vary as *T*^−1.72^ and *T*^−1.94^ for n-type and p-type material respectively. Likewise, the plot of Seebeck coefficient against ln*T* should be approximately linear with a slope of 3*k*/2*e* or 129 μV/K whereas the actual slope is somewhat greater, for example 150 μV/K for n-type samples. However, when it comes to calculating the temperature variation of the power factor α^2^σ, for a given reduced Fermi energy, these deviations from the simple theory approximately cancel one another. This is evident from the data given in Table 1.

In Table 1 the electrical conductivities at 150 K and 300 K of n-type and p-type bismuth telluride are given for constant values of the Seebeck coefficient. The ratios of the conductivities at the two widely different temperatures are close to unity. It is, therefore, a good approximation to state that the electrical conductivity for a given Seebeck coefficient is independent of the temperature. Our prediction that *ZT* should be approximately proportional to *T* should hold for bismuth telluride alloys provided that there is only one type of charge carrier.

### 2.2. Region of Mixed Conduction

It is a different matter when we enter the temperature range for which minority carriers have to be considered. The energy gap [15,16] of the compound Bi_2_Te_3_ at 300 K is only about 5*kT*, which means that, for typical samples, minority carriers are not entirely negligible.

Minority carriers are undesirable for two reasons. Since they have thermoelectric coefficients that are opposite in sign to those of the majority carriers, they reduce the overall Seebeck coefficient. Also, they can make a large contribution to the thermal conductivity through the bipolar effect [4].

Provided that the energy gap is not too small, the harmful minority carrier effects can be minimized by increasing the concentration of the majority carriers. This reduces the Seebeck coefficient but the increase in electrical conductivity almost makes up for this when the power factor is calculated. Increasing the doping level is clearly one way of minimizing the minority carrier effects in bismuth telluride. However, one must also take into account an increase in the single-carrier electronic thermal conductivity as the carrier concentration rises.

The maximum value of the Seebeck coefficient for a non-degenerate semiconductor is simply related to the energy gap, *E*_g_. In general, it is a good approximation [17,18] to set α_max_ equal to ±*E*_g_/2e*T* unless there is a very large ratio between the electron and hole mobilities. Thus, if bismuth telluride has an energy gap equal to 5*kT*, the maximum Seebeck coefficient is estimated to be about ±5*k/*2*e*, or ±216 μV/K. In actual fact, the Seebeck coefficient of this compound can exceed ±250 μV/K but it is not surprising that the estimate is rather crude in view of the partial degeneracy that must exist. The fact that the maximum Seebeck coefficient is so close to the optimum value for a single band indicates that minority carrier conduction is already having some influence at room temperature. There is absolutely no doubt that minority carrier conduction must be taken into account for bismuth telluride thermoelements that are designed for use at higher temperatures. This means that we must accept lower values of the Seebeck coefficient, perhaps less than ±200 μV/K, for operation at upwards of, say, 400 K.

Of course, the most obvious way of extending the effectiveness of bismuth telluride alloys to higher temperatures is by increasing the energy gap. It is likely that the energy gap in a solid solution will be different from that of either of its components. For narrow-gap semiconductors the Kane [19] model suggests that the inertial effective mass will rise with increasing gap leading to a decrease in the mobility. We might think, then, that there could be a general rule that the larger the energy gap the smaller the carrier mobility and also, perhaps, μ(m*/*m*)^3/2^, where m* is the density-of-states mass. However, we have not been able to establish a clear relationship between either μ or μ(m*/*m*)^3/2^ and the energy gap for all those semiconductors for which data were available [20]. There is some indication that the mobility falls with increasing gap but the trend is not very clear. This is somewhat surprising but it does not alter the fact that the best thermoelectric materials at any given temperature often have values of the energy gap that are no more than a few *kT*.

**Figure 2.**
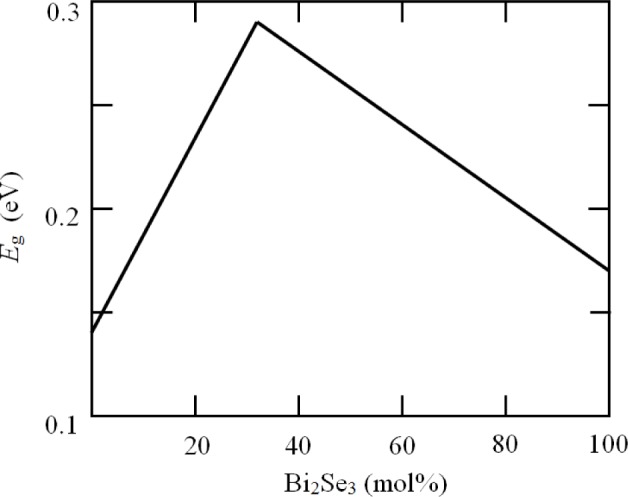
Variation of energy gap with concentration of Bi_2_Se_3_ in Bi_2_(Te-Se)_3_ alloys based on the observations of Greenaway and Harbeke [16].

There is the possibility that some of the solid solutions may have larger energy gaps than bismuth telluride itself. The variation of energy gap with composition has been studied for the Bi_2_Te_3_-Bi_2_Se_3_ system by Greenaway and Harbeke [16]. As shown in Figure 2, it appears that the energy gap rises by a factor of about 2 as *x* changes from 0 to 1 in the series Bi_2_Te_3−_*_x_*Se*_x_*. It seems [21] that the largest gap occurs when *x* = 1. This should allow minority carrier conduction to be all but eliminated up to a temperature of about 500 K. However, the electron mobility becomes smaller as Se is added and it is usual to restrict the value of *x* to no more than 0.45 in n-type thermoelements for refrigeration modules [22]. Bismuth seleno-telluride alloys are commonly used as the negative components in such modules, probably because the required concentration of donor impurities to optimize the carrier concentration is less than it would be for pure bismuth telluride or for alloys containing antimony. There has been little work done on the use of bismuth seleno-telluride alloys as p-type materials, probably because it is easier to optimize p-type bismuth-antimony telluride.

There is little evidence that the energy gap changes significantly as antimony telluride is added to bismuth telluride though, until recently, it was thought that solid solutions containing more than about 75% of Sb_2_Te_3_ must have a smaller gap width since it has been a challenge to obtain a Seebeck coefficient of even 200 μV/K for such materials. It is unusual to find a Seebeck coefficient above 150 μV/K for Sb_2_Te_3_ itself, and this could indicate that the energy gap is smaller than that of Bi_2_Te_3_. However, it now appears that this is due to the non-stoichiometry in this compound rather than a small energy gap [23]. It is difficult to add enough donor impurity to compensate for the large inbuilt acceptor concentration. Sun *et al.* [24] have shown that the Seebeck coefficient can rise to about 200 μV/K at 500 K even when it is no more than about 175 μV/K at room temperature. This suggests that the energy gap is at least as large as that of Bi_2_Te_3_. *ZT* is rather small, being only about 0.25 at room temperature, but it was found to rise to about 0.55 at 550 K. The thermal conductivity is not particularly high, so the low figure of merit is due to a rather small power factor (the electrical conductivity is much smaller than that of bismuth telluride with the same Seebeck coefficient). It is apparent from this work that alloys that are rich in Sb_2_Te_3_ may be useful generator materials. Mehta *et al.* [25] think that the energy gap of Sb_2_Te_3_ may well be greater than that of Bi_2_Te_3_. They suppressed the anti-site defects that normally exist in Sb_2_Te_3_ by doping with sulfur. As a result they were able to obtain *ZT* equal to 0.95 at 420 K.

One of the developments in recent years has been the introduction of energy filtering as a means of controlling the Seebeck coefficient. For example, it is possible [26] to improve the power factor in PbTe by doping with Tl. In this case, the energy states associated with Tl are located well inside the main energy band, and this leads to an enhanced Seebeck coefficient. It is possible that a similar effect occurs when Sn is added to bismuth telluride [27]. This may allow the Seebeck coefficient to remain high when the Fermi energy lies deep within the main band, thus minimizing the minority carrier contribution.

Another possibility, that would allow a higher Seebeck coefficient to be achieved with a narrow gap, is the introduction of ionized-impurity scattering. Ioffe [2] showed that the increase in the energy transported by the charge carriers, following the introduction of ionized-impurity scattering, could more than compensate for the reduction in the electrical conductivity in the determination of the power factor. However, the effect predicted by Ioffe would require rather precise control of the proportion of the scattering due to the impurities, and it would be of only marginal benefit in an extrinsic material. On the other hand, the effect would be much more beneficial in a mixed or near-intrinsic conductor [28]. It has been shown that the maximum Seebeck coefficient in a bismuth-like zero-gap conductor could be increased from 104 μV/K with lattice scattering to 242 μV/K with the optimized proportion of ionized-impurity scattering. Presumably, one could increase the contribution of ionized-impurity scattering by counter-doping, though it must be admitted that such scattering is not usually observed in bismuth telluride. That is because such a material has a very large dielectric constant, which shields the carriers from the coulombic field of the impurity ions.

## 3. Experimental Observations

In 1982, Goldsmid and Cochrane [29] designed a thermoelectric generator to work from a solar collector with a non-tracking concentrator. Their design made use of bismuth telluride alloys with a maximum operating temperature of 200 °C. They showed clearly that *ZT* at high temperatures could definitely be increased by raising the carrier concentration even though such heavily doped material would not be much good for refrigeration. After optimization, there was little change of *ZT* with temperature over the range 300 K to 450 K. The maximum value of *ZT* at 300 K for a p-type alloy was less than unity and could certainly be improved using nano-structuring. Nevertheless it is still probably true that the optimum Seebeck coefficient at different temperatures is likely to be little changed for a nanostructure. Goldsmid and Cochrane found that the best material for use at 200 °C should have a Seebeck coefficient of less than 150 μV/K at 300 K, and the virtue of choosing such a material is evident when we make a comparison with, say, the data of Imaduddin and Dupre [30] who studied p-type alloys in the temperature range 300 K to 600 K.

The thermoelectric properties of the alloys of bismuth telluride have been reviewed by several authors over the past few decades. One of the most comprehensive treatments is that of Scherrer and Scherrer [31,32]. It is generally accepted that these alloys are the best available materials for generation as well as refrigeration near room temperature [33]. Even when higher temperature sources are available, bismuth telluride alloys should be used at the low temperature ends of segmented thermoelements.

Although the problem of improving the composition of bismuth telluride alloys for low temperature applications has been treated successfully by Kutasov *et al.* [34], there seems to have been little attempt to optimize these materials for use at high temperatures.

Son *et al.* [35] produced a p-type bismuth telluride alloy using a sintering process. They found that they could control the grain size by adjustment of the ball milling time during the preparation of the powders. The grain size was small enough for the material to be described as a bulk nanostructure. An interesting feature of their observations is the clear demonstration of the need to optimize the doping for high temperature material. For example, the material with the highest value of *ZT* of 1.1 at 300 K had the rather low value of about 0.2 at 575 K. On the other hand, a sample with *ZT* equal to 0.5 at 300 K had a value of 0.6 at 575 K with a maximum of 0.9 at about 475 K.

The problem of optimizing the impurity content of bismuth telluride alloys for specific temperatures has been tackled by Kuznetsov [36] in the production of functionally graded thermoelements. Min and Rowe [37] discussed the optimization of Peltier modules for use in power generation but did not treat the question of the composition of the thermoelements. Matsuura and Rowe [38] selected bismuth telluride alloys as the preferred generator materials up to 150 °C but did not consider the optimization of the Seebeck coefficient. It is noteworthy, however, that their n-type alloy, Bi_2_Te_2.25_Se_0.75_, displayed an almost constant value of 2 × 10^−3^ K^−1^ for *Z* at all temperatures from 0 °C to 150 °C, which implies a continuous rise of *ZT* with temperature over this range. Dashevsky *et al.* [39] reviewed generator materials up to 800 °C, specifying Bi_2_Se_0.6_Te_2.4_ and Bi_0.5_Sb_1.5_Te_3_, as the n-type and p-type materials up to 300 °C, but without discussing the optimization of the carrier concentration.

Kusano and Hori [40] described the properties of Bi_0.4_Sb_1.6_Te_3_ with 0.3 wt% of PbTe and showed that *ZT* rose from about 1.0 at 323 K to 1.1 at 380 K and then fell to 0.8 at 623 K. The material was produced by spark plasma sintering and, presumably, did not display any nanostructure effects. It may well represent the best that can be done without nanostructuring unless the energy gap can be increased to minimize the effect of minority carrier conduction.

It has generally been found that good n-type bismuth telluride alloys are more difficult to produce than their p-type counterparts. This is in part due to the rather high ratio of the electron mobility parallel to and perpendicular to the cleavage planes. Thus, unaligned polycrystalline specimens are bound to be inferior to aligned crystals, with *ZT* closer to 0.5 than 1. Nevertheless, such polycrystalline material is attractive from the practical viewpoint. It has been usual to select n-type materials from the Bi_2_Te_3_-Bi_2_Se_3_ system, but it should not be forgotten that suitably doped samples in the Bi_2_Te_3_-Sb_2_Te_3_ system will also be n-type. Thus, the properties of n-type Bi*_x_*Sb_2−_*_x_*Te_3_, with *x* between 0.5 and 0.7, have been studied by Gerovac *et al.* [41] with a view to their use in generators. The figure of merit of these alloys is no larger than that of the Bi_2_Te_3_-Bi_2_Se_3_ alloys and, in view of their increased energy gap, the latter would appear to be the more promising for higher temperature use. It is expected that the best n-type materials will display some preferential orientation, the most practical production process, for achieving this end, involving hot extrusion [42,43].

Nanostructured bismuth telluride has been investigated at temperatures up to 430 K by Yu *et al.* [44]. The samples were prepared, by either spark plasma sintering or a high temperature—high pressure process, from hydrothermally produced nanorods. The rather low Seebeck coefficient, 130 to 150 μV/K at room temperature, suggests that the material could well be optimized for high temperature use. However, as acknowledged by the authors, their samples were inhomogeneous and there is evidence of mixed conduction from the thermal conductivity data. The low values of *ZT* between 0.4 and 0.5 do not necessarily mean that this type of processing will always yield poor material.

Good p-type material seems to have been produced by Li *et al.* [45]. Bulk nanostructured samples of a p-type (Bi-Sb)_2_Te_3_ alloy have been made by mechanical alloying and spark plasma sintering. The measurements covered the temperature range up to 580 K and included heavily doped material in which the Seebeck coefficient rose continuously with temperature. *ZT* was found to be 1.39 at 370 K so the material certainly seems suitable for use just above room temperature. However, it is noted that the acceptor impurity was copper, which has often been found to behave as a donor, when it lies between loosely bonded tellurium layers in the crystal lattice. In these interstitial positions it is a rapid diffuser even at room temperature [46]. Nevertheless, other workers [47] have also reported useful thermoelectric properties from copper- and silver-doped material.

One of the few papers to describe the properties of nanostructured material above room temperature has been published by Bulat *et al.* [48]. The material was p-type with the composition Bi_0.4_Sb_1.6_Te_3_. The high figure of merit, *ZT*, equal to 1.25 at about 100 °C, is attributed to a very small lattice conductivity. The sintered material was prepared from powders with a grain size of the order of 100 nm. The importance of the structure of the powders in any sintering process is apparent from the work of Wu *et al.* [49]. The best results were achieved using flower-like nanosheets yielding *ZT* of the order of unity over the whole temperature range 300 K to 525 K. Somewhat higher values of *ZT* were obtained by Xie *et al.* [50] who combined melt spinning and spark plasma sintering. A *ZT* value of 1.5 at 400 K was found for p-type Bi_0.5_Sb_1.5_Te_3_ but when the same techniques were applied to n-type Bi_2_(Te_1−_*_y_*Se*_y_*)_3_ they yielded a *ZT* value of only 1.0. This, of course, may be an acceptable figure but it could, no doubt, be improved by orienting the grains, perhaps by extrusion.

Most of the early studies of sintered Bi_2_Te_3_ alloys made use of large particle sizes to minimize the effect of oxidation [51]. On the other hand, very fine powders are needed in the production of nanostructured material. This problem has been tackled successfully by Nguyen *et al*. [52] using a spark erosion technique. Samples of p-type Bi_0.5_Sb_1.5_Te_3_ made by sintering these powders have yielded a *ZT* value of 1.36 at 360 K.

Non-aligned n-type material with the formula Li*_x_*Bi_2_Se_0.3_Te_2.7_ has been prepared by Chen *et al.* [53] using a sintering method. The authors claim that the use of Li as a dopant improves *ZT* by about 25%. However, the observed value of 0.8, while quite high for non-aligned material, is no better than other workers have achieved. A significant feature of this work is the maintenance of a reasonably high *ZT* value up to a temperature of 500 K.

Kim *et al.* [54] produced n-type nanocomposites of bismuth seleno-telluride by introducing Bi_2_Se_3_ nanoparticle inclusions into bulk Bi_2_Te_3_. The reduction in the lattice conductivity was reflected in a rise of *ZT* at 320 K from 0.56 to 0.75. Measurement of the thermoelectric properties covered temperatures up to nearly 600 K. However, *ZT* fell continuously with increasing temperature over the whole range. This was largely because the Seebeck coefficient was continuously decreasing. That this should have occurred with a Seebeck coefficient of no more than −150 μV/K at 320 K is entirely unexpected and may indicate non-uniformity of composition. Part of the sample could have displayed mixed conduction and part extrinsic conduction, both regions having a low Seebeck coefficient.

**Figure 3.**
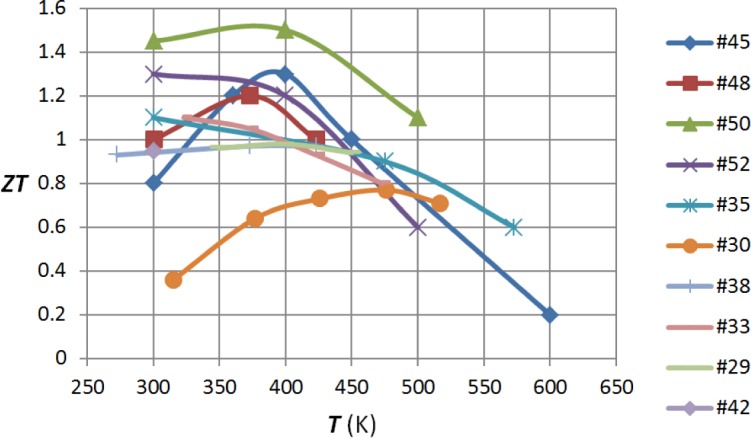
Plot of *ZT* against *T* for p-type bismuth telluride alloys. The sources of the values are the reference numbers as indicated.

**Figure 4.**
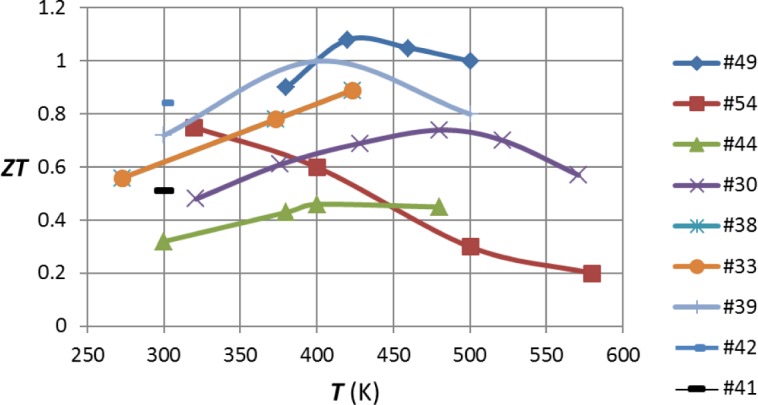
Plot of *ZT* against *T* for n-type bismuth telluride alloys. The sources of the values are the reference numbers as indicated.

Figures 3 and 4 summarize the results of the various authors that have been mentioned in this review. It must be remembered that the accurate measurement of the figure of merit is not simple, particularly at elevated temperatures. Typically [55], the error in determining this quantity may be as high as 20%. Thus, the highest and lowest values in these diagrams may not respectively indicate exceptionally good or bad material.

## 4. Practical Considerations

Thermoelectric refrigerators almost invariably make use of linear flow in multi-couple modules but for thermoelectric generation other geometric configurations may be preferred. Thus, a tubular arrangement is particularly suitable for harvesting the energy from fluid heat sources. It must be remembered that one of the earliest applications of thermoelectric generation, using an oil burner as the heat source, adopted this type of arrangement [2]. However, if a tubular configuration is sought, this may be more easily achieved using the transverse rather than the longitudinal Seebeck effect [56]. A transverse thermoelement is probably best produced from a synthetic two-phase material [12]. A thermoelectric generator, using the transverse Seebeck effect in a synthetic transverse material comprising p-type Bi_0.5_Sb_1.5_Te_3_ and Ni, has been described by Sakai *et al.* [57]. The figure of merit would have been close to that of a longitudinal (Bi-Sb)_2_Te_3_—Ni thermocouple. Other combinations would probably be superior, perhaps with the nickel being replaced by an n-type conductor such as bismuth, though Kyarad and Lengfellner [58] used a metal, lead, in conjunction with a bismuth telluride in their transverse device.

Thermoelectric modules are usually made from n-type and p-type thermoelements joined by metallic bridges. Whether or not such bridges are employed, it is necessary to make electrical contact between the components and, for this purpose, metallization of the thermoelements and some type of solder is usually needed. A joining technique that is satisfactory for refrigeration modules may not be sufficiently stable at the elevated temperature in a generator. Li *et al.* [59] have shown that antimony is a suitable contact material for both bismuth-antimony telluride and bismuth seleno-telluride. The problem of contacts was solved by Kadhim *et al.* [60] using silver paste in their generator operating from a heat source at 523 K.

The stability of thermoelectric generators over long time periods is probably an essential requirement. Tellurium is a volatile element and its loss would severely affect the thermoelectric properties of the alloys. In this context, the use of protective coatings may be necessary. Tests have been made by Brostow *et al.* [61] on polymer coatings and it has been found that weight loss has then been prevented at temperatures of up to 500 °C. Ceramic coatings cracked under the same conditions.

For the time being, most experimental thermoelectric generators [62], that operate at relatively low temperatures, will make use of existing modules, perhaps selected from batches that have low Seebeck coefficients. Test systems for such modules, under the conditions at which the generators will be operated, have been developed [63].

The question of the availability of the component elements has not been addressed. In this context it may be noted that the cost and scarcity of tellurium may make alloys based on bismuth telluride unsuitable for large scale applications.

## 5. Conclusions

It has been firmly established that *ZT* at 300 K has a maximum value of 1.0 for large-grained p-type (Bi-Sb)_2_Te_3_ with a substantially higher value for nanostructured material. *ZT* is almost as high at 0.9 for aligned n-type Bi_2_(Se-Te)_3_ though a value closer to, say, 0.6 is more typical for randomly oriented polycrystalline samples. By preparing samples with an increased carrier concentration and, therefore, a lower Seebeck coefficient, *ZT* can be maintained at similar values up to about 100 °C with a gradual decrease at still higher temperatures.

An increase of the energy gap would give some benefit at room temperature and would enable substantial improvements to be made above 300 K. It seems to be well established that *E*_g_ is greater for Bi_2_(Se-Te)_3_ than for Bi_2_Te_3_, so minority carrier conduction should be less of a problem for n-type alloys than for p-type material. It is possible that, because of the increased gap, Bi_2_(Se-Te)_3_ could also be superior to (Bi-Sb)_2_Te_3_ as a p-type generator material, provided that sufficient acceptor impurity can be accommodated. There has also been the suggestion that Sb_2_Te_3_ may have a larger energy gap than Bi_2_Te_3_ and, if this is so, (Bi-Sb)_2_Te_3_ with a reduced bismuth concentration may be preferred for generator applications. The non-stoichiometry of Sb_2_Te_3_ may prove to be an advantage in making p-type thermoelements for high temperature use.

Failing any increase in the energy gap, some energy filtering mechanism may allow the Seebeck coefficient to remain high at elevated temperatures, without appreciable minority carrier conduction. The resonant levels associated with tin in bismuth telluride and its alloys may be useful in this way, though the original studies [27] did not deal with the Seebeck coefficient as a function of temperature. Likewise, although there would undoubtedly be benefits for generator materials arising from a moderate amount of ionized-impurity scattering, experimental work to support this concept is lacking.

**Figure 5.**
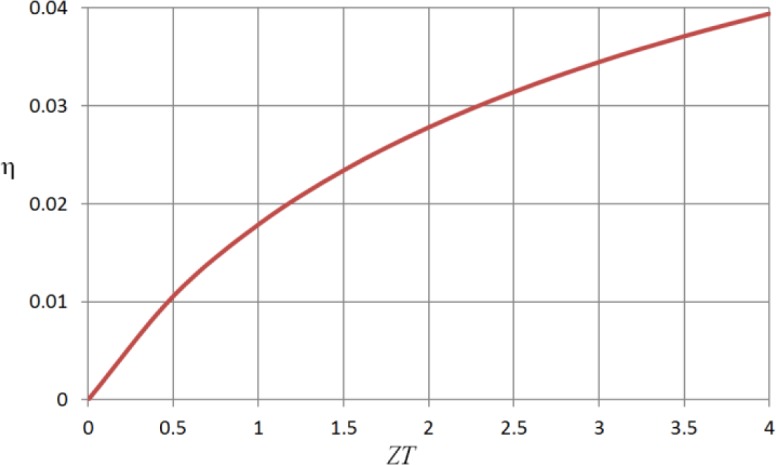
Plot of efficiency against dimensionless figure of merit for the heat source at 400 K and the heat sink at 360 K.

Figure 5 shows the variation of efficiency with *ZT* for a typical low grade heat source. The system that is envisaged would provide a modest amount of electrical power together with an ample supply of hot water. The heat source is supposed to be at 400 K and the heat sink to take the form of water at 360 K. The plot shows that a generation efficiency of about 2% should be attainable without any further material developments. The efficiency for a Carnot cycle operating between the same source and sink temperatures would be 10%. Efficiencies of the order of 20% of the Carnot cycle value are, in fact, typical for thermoelectric generators over a wide range of temperature.

In summary, it should be possible to produce both p-type and n-type bismuth telluride alloys that maintain a value of *ZT* equal to at least unity up to a temperature of 500 K, with a reasonable likelihood of obtaining significantly greater values over at least part of the temperature range. It is likely that the material will have a composition with a larger energy gap than that of bismuth telluride. Otherwise, minority carrier effects may be minimized by the addition of impurities, such as tin, with resonant states, or by the introduction of ionized-impurity scattering. Improved material is likely to have a bulk nanostructure with the n-type material displaying substantial preferred orientation, possibly achieved by an extrusion technique.

## Figures and Tables

**Table 1.** Electrical conductivity of n-type and p-type Bi_2_Te_3_. The Seebeck coefficient is −170 μV/K for the n-type material and 160 μV/K for the p-type material.

Type	σ at 150 K	σ at 300 K	Ratio of conductivities at 300 and 150 K
n	1.5 × 10^3^ Ω^−1^·cm^−1^	1.655 × 10^3^ Ω^−1^ cm^−1^	1.10
p	1.55 × 10^3^ Ω^−1^ cm^−1^	1.4 × 10^3^ Ω^−1^ cm^−1^	0.90
